# Loss of regional accent after damage to the speech production network

**DOI:** 10.3389/fnhum.2015.00610

**Published:** 2015-11-05

**Authors:** Marcelo L. Berthier, Guadalupe Dávila, Ignacio Moreno-Torres, Álvaro Beltrán-Corbellini, Daniel Santana-Moreno, Núria Roé-Vellvé, Karl Thurnhofer-Hemsi, María José Torres-Prioris, María Ignacia Massone, Rafael Ruiz-Cruces

**Affiliations:** ^1^Cognitive Neurology and Aphasia Unit and Cathedra Foundation Morera and Vallejo of Aphasia, Centro de Investigaciones Médico-Sanitarias, University of MalagaMalaga, Spain; ^2^Department of Psychobiology and Methodology of Behavioural Sciences, Faculty of Psychology, University of MalagaMalaga, Spain; ^3^Department of Spanish Language I, University of MalagaMalaga, Spain; ^4^Molecular Imaging Unit, Centro de Investigaciones Médico-Sanitarias, General Foundation of the University of MalagaMalaga, Spain; ^5^Department of Applied Mathematics, Superior Technical School of Engineering in Informatics, University of MalagaMalaga, Spain; ^6^Centro de Investigaciones en Antropología Filosófica y Cultural, Consejo Nacional de Investigaciones Científicas y TécnicasBuenos Aires, Argentina

**Keywords:** speech production, regional accent, foreign accent, motor speech disorder, stroke

## Abstract

Lesion-symptom mapping studies reveal that selective damage to one or more components of the speech production network can be associated with foreign accent syndrome, changes in regional accent (e.g., from Parisian accent to Alsatian accent), stronger regional accent, or re-emergence of a previously learned and dormant regional accent. Here, we report loss of regional accent after rapidly regressive Broca’s aphasia in three Argentinean patients who had suffered unilateral or bilateral focal lesions in components of the speech production network. All patients were monolingual speakers with three different native Spanish accents (Cordobés or central, Guaranítico or northeast, and Bonaerense). Samples of speech production from the patient with native Córdoba accent were compared with previous recordings of his voice, whereas data from the patient with native Guaranítico accent were compared with speech samples from one healthy control matched for age, gender, and native accent. Speech samples from the patient with native Buenos Aires’s accent were compared with data obtained from four healthy control subjects with the same accent. Analysis of speech production revealed discrete slowing in speech rate, inappropriate long pauses, and monotonous intonation. Phonemic production remained similar to those of healthy Spanish speakers, but phonetic variants peculiar to each accent (e.g., intervocalic aspiration of /s/ in Córdoba accent) were absent. While basic normal prosodic features of Spanish prosody were preserved, features intrinsic to melody of certain geographical areas (e.g., rising end F0 excursion in declarative sentences intoned with Córdoba accent) were absent. All patients were also unable to produce sentences with different emotional prosody. Brain imaging disclosed focal left hemisphere lesions involving the middle part of the motor cortex, the post-central cortex, the posterior inferior and/or middle frontal cortices, insula, anterior putamen and supplementary motor area. Our findings suggest that lesions affecting the middle part of the left motor cortex and other components of the speech production network disrupt neural processes involved in the production of regional accent features.

## Introduction

Regional accent (or *within-language accent*) is a manner of speaking peculiar to a location where its speakers reside (Wells, 1982; Cristia et al., 2012). Regional accent has coherent variations in phonetic, phonological, phonotactic, and prosodic information found within the standard language which allows it to be distinguished from other regional accents of the same language (Wells, 1982; Cristia et al., 2012). In general, the geographical, socio-economic, and ethnic background can be inferred by regional accent and dialect of speakers (Labov, 2006; Coupland, 2007). Traditionally, the study of regional accent and dialects pertains to the domains of applied psychology and socio-linguistics. Nonetheless, in recent years interest in studying accent has been expanded to the field of cognitive neuroscience to gain understanding of both the factors influencing accent perception (Adank et al., 2009; Bent and Holt, 2013; Trude et al., 2013; Smith et al., 2014) and neural mechanisms (Adank et al., 2012; Goslin et al., 2012; Callan et al., 2014). Interest in the analysis of accent in healthy subjects, however, is unbalanced since most studies have examined accent perception (Anderson-Hsieh and Koehler, 1988; Clarke and Garrett, 2004; Bradlow and Bent, 2008; Adank et al., 2009; Brunellière et al., 2009; Cristia et al., 2012; Goslin et al., 2012; Bent and Holt, 2013; Trude et al., 2013; Smith et al., 2014) rather than its production (Harrington et al., 2000; Golestani and Pallier, 2007; Golestani et al., 2007; Ventura-Campos et al., 2013). These studies have provided compelling evidence that understanding messages intoned with a non-native accent (*foreign accent*) entails more difficulty than processing regional accents, although non-familiar regional accents could also reduce the intelligibility, efficiency, and accuracy of linguistic processing (Floccia et al., 2006; Cristia et al., 2012; Van Engen and Peelle, 2014). Differences in comprehension of foreign and familiar regional accents have been interpreted as resulting from perceptual distance or different neural processing mechanisms (see Goslin et al., 2012).

Speaking with a foreign accent or unfamiliar regional accent implies a processing cost on the listener as these accents impose additional cognitive support to optimize intelligibility, comprehensibility, and speed of processing (Floccia et al., 2006; Adank et al., 2009; Bent and Holt, 2013; Van Engen and Peelle, 2014). Speaking with a non-native or unfamiliar regional accent also carries negative connotations and even jeopardizes the credibility of the speaker (accent discrimination; Lippi-Green, 1994; Lev-Ari and Keysar, 2010; Akomolafe, 2013). There are some dramatic examples; for instance, during the dictatorship of Rafael L. Trujillo (1891–1961) in the Dominican Republic, the Nobel Prize laureate Mario Vargas Llosa narrates the story of the parsley massacre (Vargas Llosa, 2000). To rapidly identify the nationality of Haitians, Dominican soldiers would hold up a sprig of parsley and asked “What is this?” Assuming that those who were incapable of pronouncing correctly the /r/ of the Spanish word “perejil” (parsley) were Haitians, soldiers assassinated more than 20000 refugees living within the Dominican border using their pronunciation as sufficient condemnation.

Interest in the study of accent change in pathological conditions is not new (Marie, 1907; Pick, 1919). Investigations on abnormal accent change have mostly been done in brain damaged individuals displaying a rare condition termed *foreign accent syndrome* (FAS; Whitaker, 1982). FAS is a motor speech disorder characterized by the development of speech patterns which are perceived as foreign (Marie, 1907; Pick, 1919; Monrad-Krohn, 1947; Whitaker, 1982; Berthier, 1994). Although the term “foreign” is generically used to designate the origin of accent change, it is noteworthy that in the original aphasic patient described by Marie (1907) speech changes occurred in “regional” accent (from Parisian French to Alsatian accent). Since then this case has been considered the original description of FAS (Whitaker, 1982) although subsequent cases of regional accent change (e.g., from native southern Ontario accent to Canadian east coast accent – Naidoo et al., 2008) have also been described (Critchley, 1962; Seliger et al., 1992; Dankovičová et al., 2001; Kwon and Kim, 2006; Ryalls and Whiteside, 2006) and re-classified as variants of FAS (Seliger et al., 1992; Verhoeven and Mariën, 2007) or foreign accent-like syndromes (Reeves and Norton, 2001). The analysis of changes in production or reception of accents using lesion-symptom mapping is providing fruitful insight on the linguistic, behavioral, and neural mechanisms underpinning the production of foreign and regional accents. These include studies in neurological patients with focal lesions (Kurowski et al., 1996; Blumstein and Kurowsky, 2006) or during the early stages of degenerative conditions (Alzheimer’s disease, primary progressive aphasia; Luzzi et al., 2008; Hailstone et al., 2012; Fletcher et al., 2013; Paolini et al., 2013).

**FIGURE 1:**
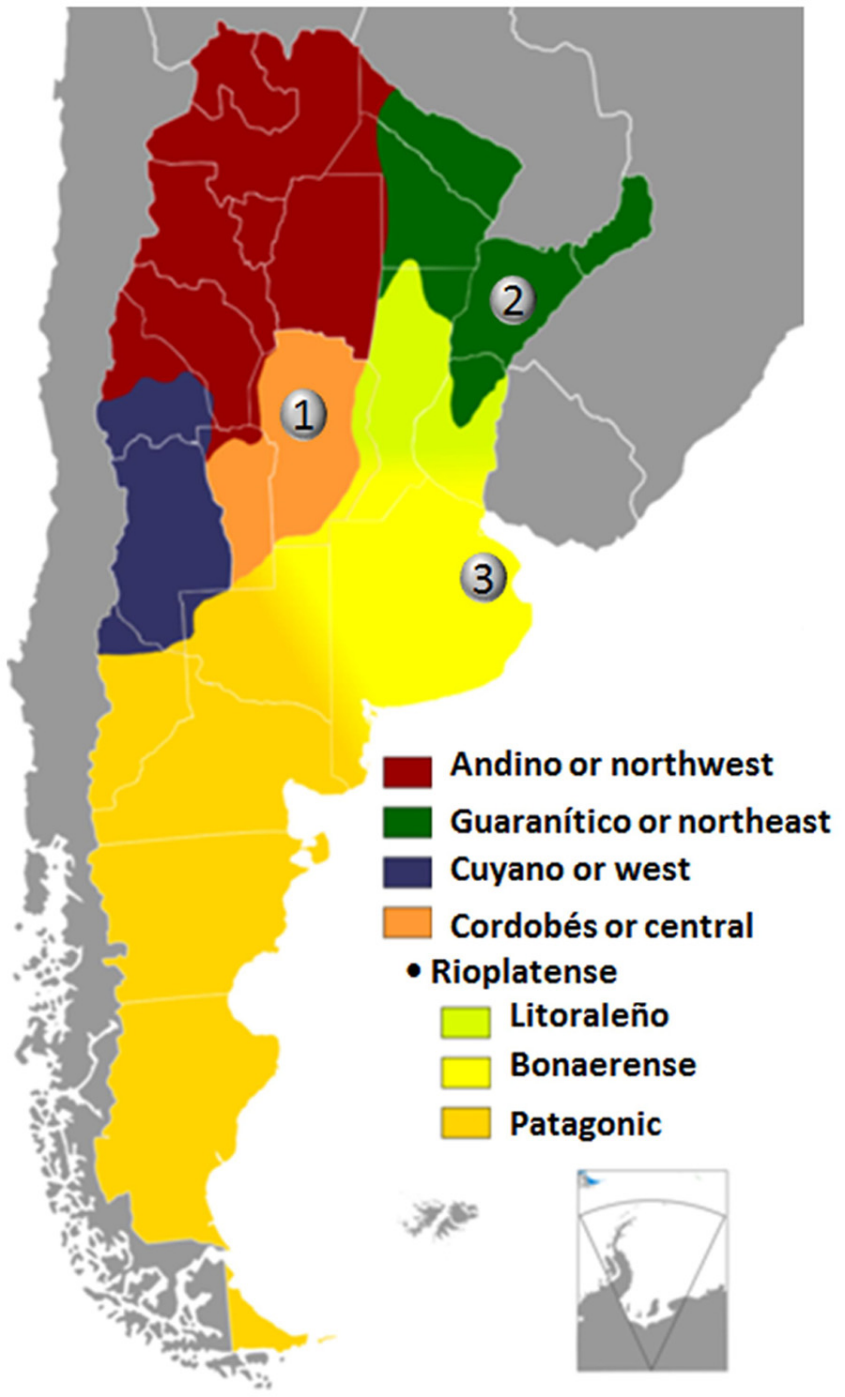
**Map of Argentina showing regional accents depicted in different colors (source http://es.wikipedia.org/wiki/Acento_cordobés), based on descriptions from Vidal de Battini (1964).** Before brain damage every patient had regional accent of the cities indicated with numbers in the map, (1) Córdoba, (2) Corrientes, and (3) Buenos Aires. The regional accent used in Córdoba is designated as Cordobés or central; the regional accent used in Corrientes is generically termed “Guarínitico” (due to the strong influence of Guaraní, one of the two languages spoken in the neighbor country Paraguay) or northeast; and the regional accent characteristic of Buenos Aires which is known as Rioplatense – bonaerense. Rioplatense makes reference to the Río de la Plata (Silver River) and Bonaerense to the city of Buenos Aires.

Perhaps because potential changes in accent are masked by co-occurring aphasia (Critchley, 1962), the status of regional accent in patients with left hemisphere damage is largely unexplored. Hence, the study of more pure cases of accent change in patients lacking prominent aphasic deficits is ideal for linguistic analysis and lesion-symptom mapping. In this context, one issue that has received little attention up to now makes reference to the loss of the segmental and suprasegmental features that characterize regional accents. Alexander et al. (1987) succinctly described the case of a person with aphasia who had lost his dense Bostonian accent as a result of a small infarct in the deep white matter near to the left anterior capsular-putaminal region. This patient had a transient impairment of prosodic production yet the loss of regional accent was persistent. Here, we report loss of regional accent after rapidly regressive Broca’s aphasia in three Argentinean patients who had suffered unilateral or bilateral focal lesions involving different components of the speech production network. All patients were monolingual speakers with three different native Spanish accents (Cordobés or central, Guaranítico or northeast, and Bonaerense; **Figure 1**).

The three patients reported here were studied almost three decades ago in Buenos Aires by two of us (MLB and MIM) when knowledge of the linguistic and neural underpinnings of pathological changes in accent were emerging (Graff-Radford et al., 1986; Blumstein et al., 1987; Gurd et al., 1988). The aim of the present study is to interpret our own data in the light of new discoveries about accents and neuroscience. In spite of the time elapsed between evaluation and the present report, our patients were studied with comprehensive methodology and we trust that due to the lack of previous similar cases the description of these cases continues to be interest. In the past few years, modern neuroimaging studies (Wise et al., 1999; Sörös et al., 2006; Eickhoff et al., 2009; Ackermann and Riecker, 2010) and computational models (Guenther et al., 2006; Bohland et al., 2010) have identified the large-scale bilateral network that mediates speech production and monitoring. Advances in the interpretation of modern cases of FAS within this neuronatomical and computational framework (Fridriksson et al., 2005; Katz et al., 2012; Moreno-Torres et al., 2013; Tomasino et al., 2013) have led to the conclusion that virtually all cases with purported changes in accent result from discrete and selective involvement of one or more components of the speech production network. This means that different clinico-pathological correlations in FAS are possible, thus FAS could be deemed heterogeneous when clinical presentation is the focus of analysis. Damage to different nodes of the speech production network can induce FAS (Graff-Radford et al., 1986; Blumstein et al., 1987; Mariën and Verhoeven, 2007), paradoxically resolve it (Cohen et al., 2009; Bhandari, 2011), trigger changes in regional accent (e.g., from Parisian French to Alsatian accent; Marie, 1907; Ryalls and Whiteside, 2006; Naidoo et al., 2008; Polak et al., 2013), awake a previously learned and dormant regional accent (Critchley, 1962; Roth et al., 1997), make regional accent stronger only in one language in polyglots (Levy et al., 2011), or even produce a FAS restricted to one language in bilinguals (Avila et al., 2004). From a nosological viewpoint, FAS is also a heterogeneous condition as several types (neurogenic, psychogenic, developmental, and mixed) have been identified (see Reeves and Norton, 2001; Mariën et al., 2006, 2009; Reeves et al., 2007).

## Materials and Methods

### Participants

#### Patient OM

A 47-years-old, right-handed man suddenly noticed speech disturbances that rapidly progressed to mutism associated to right side weakness involving the face, arm, and leg. In the first 2 days post-onset, he was able to phonate but not to produce words. This situation lasted for 20 days until he was able to utter isolated words and the name of two neighbors with normal volume but monotonous voice. By that time, his auditory and written comprehension was normal, but he was unable to repeat words and sentences and displayed crying outbursts when unable to communicate verbally. Writing was moderately impaired and voluntary bucco-facial movements were abnormal. A computerised tomography (CT) scan revealed bilateral hemorrhages involving the left motor cortex and right insula-putamen region probably resulting from untreated hypertension (Sörös et al., 2013) or less probably from sporadic cerebral amyloid angiopathy (Samarasekera et al., 2012). His relatives reported that 1 year before the present episode, OM was admitted to hospital for 2 days for dizziness, instability, and impaired handwriting that lasted 2 days, but he did not present speech or language problems. He was referred for the present language evaluation 8 months after stroke onset. Neurological examination revealed a complete recovery of the right hemiparesis and improvement of language impairment. However, his discourse was ungrammatical and contaminated by occasional instances of mitigated echolalia (incorporation of part of the questions into his responses). He had moderate reading impairment, but writing was normal. His main complaint was a change in his speech. He commented that “Now, I don’t speak as before…… my voice has changed….my language is smooth and flat and at times words come close together.” He also reported that his verbal emissions were slow and devoid of emotional coloring, and reported problems to signal emphasis in interrogative sentences. OM was a monolingual Spanish speaker, born in Córdoba (Argentina). Before the stroke he spoke with a strong regional accent which changed afterward. Nevertheless, the origin of his newly acquired accent was puzzling. OM considered that after the stroke his accent sounded Italian and 7 months after onset when he attended his father’s funeral, some relatives thought that he was speaking with Italian accent similar to the one used by his Italian father. However, his attending speech pathologist thought instead that OM had actually lost his previously regional accent. OM was a former soccer coach and at the time of the stroke he worked as a taxi driver. He suffered symptoms of mild depression and decreased motivation after the stroke.

#### Patient JF

An 18-years-old right-handed male was admitted to the emergency room with a 2 week history of fever, headaches, and vomiting. Upon admission, he developed focal seizures affecting the right face and tongue with secondary generalization and in one occasion transient speech arrest was documented after a seizure. CT and magnetic resonance imaging (MRI) scans revealed a mature, encapsulated abscess involving the left sensorimotor cortex with mass effect over the insular cortex and basal ganglia and perilesional oedema. The cerebral abscess was surgically evacuated. After surgery the patient was mute and aphonic, and had swallowing problems. The remainder of the neurological examination also disclosed tongue deviation to the right, “pseudoperipheral” right facial and velum paresis secondary to an incomplete anterior opercular dysfunction (Foix-Chavany-Marie syndrome; Starkstein et al., 1988; Martino et al., 2012). He also had a mild right hemiparesis affecting the arm with spared sensation. There was no bucco-facial apraxia. Two weeks after surgery, he regained fluent speech and at that time his mother and an aunt reported that he began to speak with a strange accent that resembled Japanese. This newly acquired accent spontaneously remitted in a few days and according to the same relatives, JF’s speech was then “flat” in several contexts. For instance, when he asked a question it was not possible to discern if he was actually asking something or not because he could not modulate intonation properly. The same thing happened when he was asked to impart angry intonation in sentences unless he was really irritated. He was referred for the present language evaluation 4 months after surgery. JF was a monolingual Spanish speaker with very limited knowledge of Brazilian Portuguese and English. He was born in Corrientes (Argentina) and lived there until 8 months before developing the cerebral abscess when he moved to Buenos Aires to study computational engineering. Before the brain lesion, JF and his mother considered that he had a typical regional accent of Corrientes.

#### Patient RC

A 54-years-old man noticed inability to manipulate objects with his right hand just before developing a generalized seizure with transient loss of consciousness. On recovering consciousness he was mute and had right facial weakness. An emergency CT scan disclosed a hemorrhage in the left frontoparietal region secondary to the rupture of a frontal arteriovenous malformation (AVM). The hemorrhage was surgically evacuated and some feeding vessels of the AVM were occluded. During surgery it was confirmed that the premotor, motor, and sensorimotor cortices were damaged. After surgery, RC awoke with a right hemiparesis (it lasted 15 days) and mutism but he demonstrated preserved auditory comprehension. Recovery of speech production was gradual and his spontaneous speech was slow and monotonous with impaired grammar, naming, and writing. In the ensuing months, fluency continued to improve and word finding difficulties decreased. By that time, his speech was also monotonous to the extent that he was unable to signal proper tone in interrogative sentences. He was referred for the present evaluation 7 months after stroke onset. On the initial interview, RC could not convey emotion through words; he frequently said “the emotion was only noticed in my face….my speech was flat…it did not have inflections.” By contrast, he made no comments on the loss of his premorbid regional accent, suggesting he was unaware of any change in accent. RC was forced as a child to write with the right non-dominant hand but he used the left hand for other activities. RC was a monolingual Spanish speaker, born in Buenos Aires (Argentina) and lived there until early adulthood. Although he had lived in other provinces (Salta and Jujuy) of Argentina during several years, he and his wife considered that he had a typical regional accent of Buenos Aires (see below) which was the place where he lived for the two decades previous to his stroke.

#### Cognitive and Language Assessment

In all patients, general intelligence was assessed with the Wechsler Adult Intelligence Scale-Revised (Wechsler, 1988), whereas non-verbal intelligence was tested with the Raven’s Colored Progressive Matrices Test (Raven, 1965; see also Kertesz, 1982). Language competence was examined with the oral subtests (spontaneous speech, comprehension, repetition, and naming) of the Western Aphasia Battery (WAB; Kertesz, 1982) and an Aphasia Quotient (AQ) was obtained to rate the severity and type of aphasia. The WAB-AQ considers that patients have aphasia when they score <93.8 (range: 0–100) and lower scores indicate more severe aphasic deficits. By contrasts, patients performing above this cut-off score (≥93.8) may actually have speech and subtle language deficits but they are not classified as having clinically significant aphasia. The Token Test (TT; De Renzi and Vignolo, 1962) was also administered to all patients. The TT is designed to assess verbal comprehension of commands of increasing complexity. The test employs a set of 20 plastic tokens consisting of two shapes (circles and squares) depicted in five colors and two sizes (small and big). The long version of the TT (62 items) was used for the present study. Phonological fluency was assessed with the Controlled Oral Word Association Task (COWAT; Borkowski et al., 1967). The study was performed according to the Declaration of Helsinki and the protocol was approved by the Ethical Committee of the Raúl Carrea Institute for Neurological Research (FLENI), Buenos Aires, Argentina. All patients provided written informed consent prior to the detailed analysis of their speech-language deficits.

#### Lesion Analysis

Lesion location in CT and MRI scans were examined by a neuroradiologist (RRC) blind to clinical information. Identification of damaged areas and the limiting fissures and sulci was performed using an atlas of neuroanatomy of language regions of the human brain (Petrides, 2014) and a brain atlas based on ultra-high field (7.0 Tesla) *in vivo* MRI and cadaver cryomacrotome sections (Cho et al., 2015). Identification of subregions in the motor cortex for phonation and articulation, including lip, tongue and jaw areas, was based on images of previous brain imaging studies (Fesl et al., 2003; Naidich et al., 2004; Brown et al., 2008; Grabski et al., 2012). Although two patients (OM and RC) had lesions in more than one region of the speech production network, the principal component of lesions in every patient overlapped in the left pericentral region. Thus, those parts of the lesions involving the premotor, precentral, and post-central areas were manually traced by one member of the team (NRV) and verified for reliability by two members (RRC and KTH) with experience in neuroradiology. Since the lesion (cerebral abscess) in JF had marked mass effect and perilesional oedema, lesion analysis in this patient was done in a control CT scan performed 3 months after the surgical removal of the abscess. Lesions were drawn on representative axial T_1_-weigthed MRI axial templates from the MRIcron software (Rorden, C., 2005. www.mccauslandcenter.sc.edu/mricro/mricron/). Lesion overlap in the left premotor and motor cortex was carried out with Imcalc from the Statistical Parametric Mapping (SPM) software package version 8 (Welcome Department of Cognitive Neurology, London, UK). The identification of involved cortical areas was done using the Automatic Anatomical Labeling (AAL) atlas (Tzourio-Mazoyer et al., 2002). All anatomical regions identified by AAL were verified by comparing with anatomical atlases of MRI (Petrides, 2014; Cho et al., 2015).

#### Analysis of Accent

Loss of regional accent in our three patients was studied through acoustic analysis, both segmental and prosodic, to determine the phonetic characteristics which distinguish this disturbance from typical regional accent. The absence of experimental data about regional accent in Argentinian’s speakers of Spanish leads us to take into account the few impressionistic observations found in the literature (Vidal de Battini, 1964; Fontanella de Weinberg, 1971) when analyzing the data. In patient OM, a within-subject study was possible because a recording of his voice previous to the stroke was available. In the two remaining patients, regional accents were compared with data from healthy controls. In the case of patient JF, his emissions were compared with data obtained from a gender-, age-, and original dialect-matched control speaker. Data from patient RC were compared with the results obtained from four healthy control subjects who had participated in the assessment of Buenos Aires Spanish (Manrique, 1980; Manrique and Signorini, 1983; Massone, 1988).

#### Segmental Analysis

The study of both segmental and prosodic analysis utilized a sentence-reading procedure whereby speakers were asked to read eight declarative sentences that had been constructed to include particular variation in syntactic structure. A sample of spontaneous speech was used for segmental analysis. A set of sentences from the mood and tonic accent test were also included. The stimuli used for the analysis were recorded in a sound-treated room on an Ampex AG 440-2 tape recorder. Narrow phonetic transcriptions of this material were performed by two trained phoneticians. The acoustic description was accomplished with narrow and wide-band spectrograms extracted by mean of a Kay Elemetrics 7029. The following parameters were measured in the spectrograms: formant frequencies, voice-onset time (VOT) in voiceless stops, frequency position of noise bands and segment duration.

#### Prosodic Analysis

The prosodic patterns were examined in F0 plots which were obtained from a computer program based on the FPRD (fundamental period; Cooper and Sorensen, 1981) run on a PDP 11 Digital computer. The following measurements were made on the plots for each utterance: (1) initial and final F0 values; (2) the contours were analyzed according to the subcontours each had. The highest and the lowest F0 values in each subcontour were measured as the peak and valley of that subcontour. The subcontour starts on the syllable carrying an F0 accent and includes all following unaccented syllables. The overall average F0 was obtained by averaging the values of peaks and valleys. (3) The top reference line was adopted (O’Shaughnessy, 1976; Cooper and Sorensen, 1981) in order to describe the F0 general pattern of declination in declarative sentences. These lines were traced across the F0 peak values. The rate of declination (Hz/sec) was measured from the top line, which is the result of connecting the F0 peaks of the utterance. (4) For each contour, the F0 variation was defined as the difference between the F0 values for the lowest valley and for the highest peak. The range of F0 variation was the mean across the different contours.We also adopted an adjusted range measurement to counterbalance the effect of extreme points, by extracting the average values of all peaks (Ryalls et al., 1987); (5) Duration in msec of syllabic types (CV, CVC, CVVC) in stressed, unstressed and prepausal conditions was also measured. (6) Inter-stress intervals were measured from the consonant/vowel onset of the syllable bearing the stress to the consonant/vowel onset of the next syllable bearing stress; (7) speech rate was measured based on the duration of the inter-stress intervals and the duration of utterances. The healthy control subjects underwent the same assessment procedure.

#### Prosodic Tests

All patients reported problems producing emotional intonation to sentences. This finding coincides with description from previous cases of FAS and linguistic and affective dysprosody (Berthier et al., 1991; Moreno-Torres et al., 2013). Therefore, a mood production task (Weintraub et al., 1981; Graff-Radford et al., 1986; Berthier et al., 1991) was used. The three patients were asked to read four sentences with angry, sad, happy, neutral, and interrogative intonation. Targets sentences had a neutral propositional message (e.g., “Mañana voy a viajar a Mendoza” →*“Tomorrow I’m leaving for Mendoza”*). Perceptual judgements of affective and linguistic prosody production were rated by six undergraduate students of phonetics blind to patients’ identity in the five different categories. F0 curves were generated from these recordings. Raters reported no speech or hearing problems, and none of them had extensive experience in rating pathological voices. The raters were required to assign one of the four affective tones (happy, angry, sad, neutral), and one linguistic tone (interrogative) to each sentence produced by the patients. Patients were also asked to place tonic accents in sentences following the methodology described in previous studies (Weintraub et al., 1981; Graff-Radford et al., 1986; Berthier et al., 1991). Based on visual analysis of F0 increases, the expert phoneticians decided whether or not the emphatic stress was corrected signaled. Patient heard a series of 10 declarative sentences (e.g., “Los anteojos están encima de la mesa” “The glasses are on the table”) and were then asked some questions (e.g., ¿Qué está encima de la mesa? “What is on the table?”; “¿Dónde están los anteojos?” “Where are the glasses?”). Patients were informed that they should answer these sentences by placing emphatic stress on the appropriate words (subject, object, verb) depending on the questions. The full set was composed of 25 questions (10 base questions with two or three tonic accents each). Here, the patients were asked to mark the emphasis on the appropriate words by using appropriate word stress. Comprehension of affective prosody was also examined (Ross, 1981). Patients listened to 16 tape-recorded sentence intoned with angry, happy, sad and neutral intonation and were asked to identify the imparted intonation in a multiple choice format.

## Results

### Cognitive and Language Functions

General intellectual functions were preserved with average or above-average intellectual quotients scores (**Table 1**). Non-verbal intelligence was also normal in the two patients tested (OM and RC; **Table 2**). On the WAB, all patients obtained AQs well-above the cut-off scores for diagnosis of aphasia. All patients had fluent and very informative spontaneous speech with no word finding difficulties or agrammatism. Although auditory comprehension of complex sentences (sequential commands subtest) was still mildly impaired in patients OM and RC, their performance on the TT was normal, as was patient JF’s performance. Semantic category (animal naming) word generation though impaired in all patients was less affected than phonemic fluency (COWAT). Reading was mildly impaired in patients OM and RC and writing was additionally compromised in OM. Reading and writing could not be tested in patient JF.

**Table 1:** Scaled scores from the WAIS.

Wechsler Adult Intelligence Scale	OM	JF	RC
Verbal scale			
Information	6	13	13
Comprehension	6	14	12
Arithmetic	13	15	10
Similarities	10	16	14
Digit span	7	11	9
Vocabulary	8	12	11
Verbal IQ	92	123	111
Performance scale			
Digit symbol	6	8	6
Picture completion	9	11	9
Block design	11	15	11
Picture arrangement	11	8	8
Object assembly	11	13	5
Performance IQ	109	103	97
Full-IQ	99	116	105

**Table 2:** Language and cognitive functions.

Tests	OM	JF	RC
Western Aphasia Battery (WAB)			
* Spontaneous Speech*			
Information content (maximum: 10)	10	10	10
Fluency (maximum: 10)	10	10	9
* Comprehension*			
Yes/no Questions (maximum: 60)	57	58	57
Auditory Word Recognition (maximum: 60)	55	60	60
Sequential Commands (maximum: 80)	66	80	72
Total (maximum: 10)	8.9	8.9	9.45
* Repetition* (maximum: 10)	9.6	10	9.8
* Naming*			
Object Naming (maximum: 60)	57	58	60
Word Fluency (maximum: 20)	11	17	10
Sentence Completion (maximum: 10)	8	10	10
Responsive Speech (maximum: 10)	19	10	10
Total (maximum: 10)	9.4	9.5	9.0
* Aphasia Quotient* (maximum: 100)	95.8	96.8	94.5
WAB – Reading (maximum: 100)	87	NT	71
WAB – Writing (maximum: 100)	86	NT	96
WAB – Praxis (maximum: 60)	59	60	60
WAB – Construction			
Drawing (maximum: 30)	29	NT	29
Block design (maximum: 9)	9	NT	9
Calculation (maximum: 24)	24	NT	24
Raven Colored Progressive Matrices (maximum: 37)	34	NT	34
Token Test (maximum: 62)	60.5	58	60
Controlled Oral Word Association Task	7	6	6

*NT indicates not tested.*

### Neuroimaging

**FIGURE 2:**
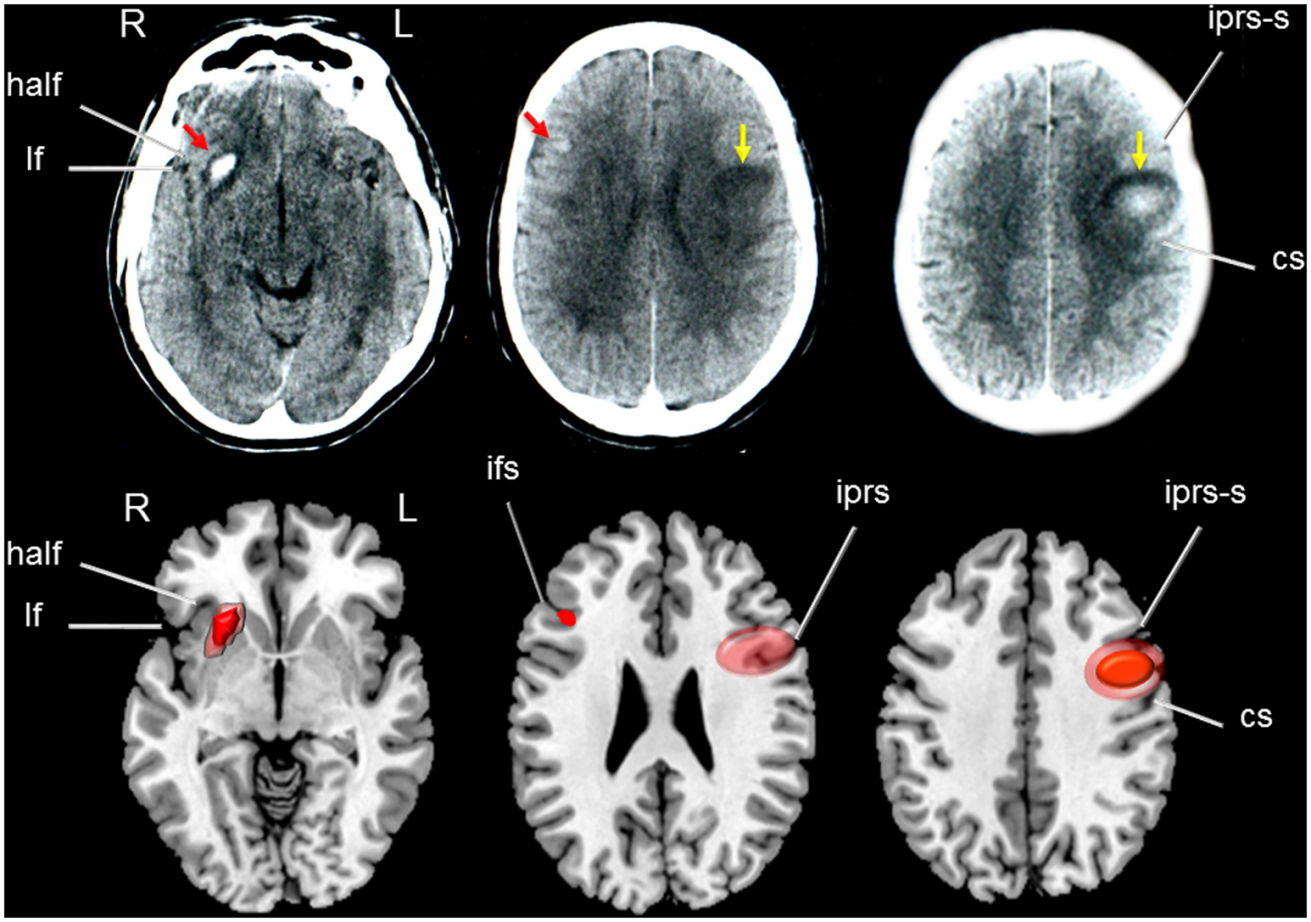
**(Top)** Axial high resolution CT scan of patient OM showing a small hemorrhage involving the right anterior putamen and insula with dorsal extension to the inferior frontal gyrus (red arrows in left and middle images) surrounded by mild vasogenic oedema. Yellow arrows indicate a round image of mixed density in the left sensory-motor regions consistent with a resolving hemorrhage. **(Bottom)** Drawings were made using MRIcro software (Rorden, C., 2005. www.mccauslandcenter.sc.edu/mricro/mricro/) on T_1_-weighted axial images. Areas depicting dense hemorrhages are drawn in red and areas of mixed density (hemorrhages and oedema) in light red. Fissures and sulcus are indicated with white arrows. half indicates: horizontal ascending ramus of the lateral fissure; lf: lateral fissure; iprs-s: inferior precentral sulcus, superior ramus; cs: central sulcus (sulcus of Rolando); ifs: inferior frontal sulcus; iprs: inferior precentral sulcus. Terminology and abbreviations for fissures and sulci were taken from the atlas of neuroanatomy of language regions of the human brain (Petrides, 2014). R: indicates right and L: left.

**FIGURE 3:**
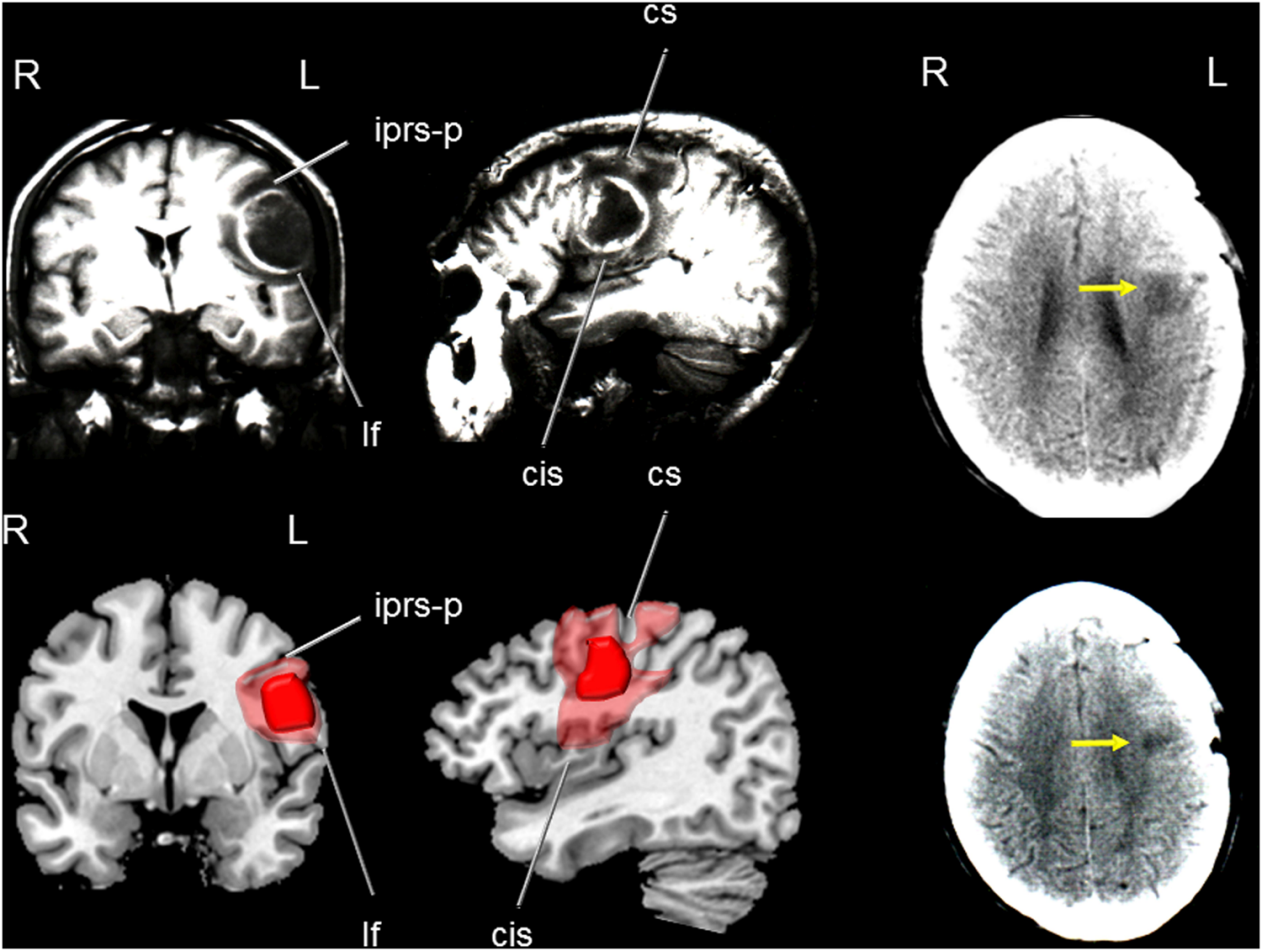
**(Top)** Coronal (left) and parasagittal (medial) T_1_-weighted MRI of patient JF showing a rounded, mature and encapsulated cerebral abscess involving the left sensory-motor cortex (medial part of the precentral gyrus) surrounded by vasogenic oedema. Note in the coronal view mass effect over the insula and basal ganglia. **(Bottom)** Drawings were made using MRIcro software (Rorden, C., 2005. www.mccauslandcenter.sc.edu/mricro/mricro/) on T_1_-weighted axial images. The abscess is drawn in red and areas of vasogenic oedema in light red. CT images performed 3 months after surgery (right images in the upper and lower panels) show a hypodense lesion in the left precentral gyrus. Fissures and sulcus are indicated with white arrows. iprs-p indicates: inferior precentral sulcus- posterior ramus; lf: lateral fissure; cs: central sulcus (sulcus of Rolando); and cis: central insular sulcus. Terminology and abbreviations for fissures and sulci were taken from the atlas of neuroanatomy of language regions of the human brain (Petrides, 2014). R: indicates right and L: left.

**FIGURE 4:**
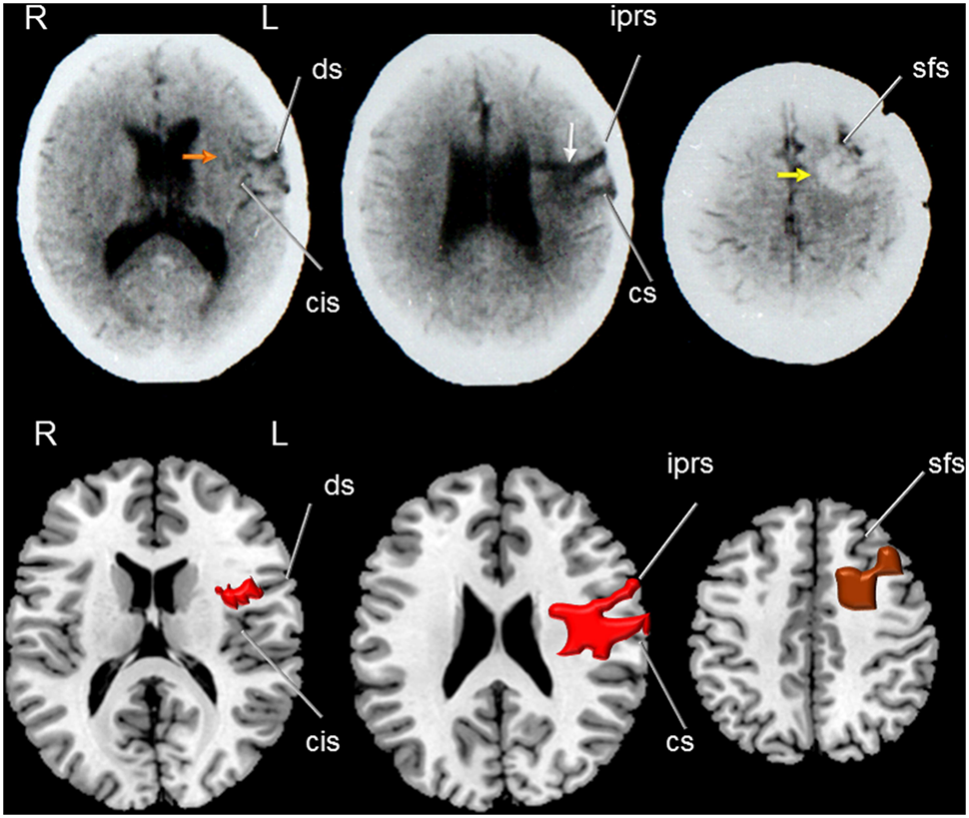
**(Top)** Axial CT of patient RC showing a hypodense lesion (middle image) involving the middle part of the left precentral gyrus (white arrow) and extending deeply to dorsal head of the caudate nucleus. Note in the left image the involvement of the anterior insula (red arrow) and shrinkage of the middle and posterior insular cortex (orange arrow) and part of the inferior precentral gyrus and superior temporal gyrus. The right image shows a hyperdense rounded image which corresponds to a remnant of the arterio-venous malformation (AVM) involved the left superior frontal gyrus reaching the pre-supplementary motor area and the subcortical white matter (anterior centrum semiovale; yellow arrow). **(Bottom)** Drawings were made using MRIcro software (Rorden, C., 2005. www.mccauslandcenter.sc.edu/mricro/mricro/) on T1-weighted axial images. The lesion is drawn in red (left and middle images) and the AVM in brown (right image). Fissures and sulcus are indicated with white arrows. ds indicates: diagonal sulcus; cis: central insular sulcus; iprs: inferior precentral sulcus inferior; cs: central sulcus (sulcus of Rolando); and sfs: superior frontal sulcus. Terminology and abbreviations for fissures and sulci were taken from the atlas of neuroanatomy of language regions of the human brain (Petrides, 2014). R: indicates right and L: left.

A CT scan in OM revealed a resolving hemorrhage in the middle part of the left motor cortex surrounded by mild oedema and mass effect over the insular cortex and anterior corona radiata. There also was a small hemorrhagic focus involving the right anterior putamen, anterior insula, and dorsal part of the inferior the frontal gyrus (**Figure 2**). CT and MRI scans in JF revealed a mature, encapsulated abscess involving the left sensorimotor cortex surrounded by vasogenic edema and mass effect over the insular cortex and basal ganglia. Three months after surgery a control CT scan showed a small residual lesion involving the left primary motor area (**Figure 3**). In RC, a CT performed in the chronic period showed a hypodense lesion involving the left lower portion of the precentral gyrus and its adjacent pars opercularis of the inferior frontal gyrus, anterior insula, and superior temporal gyrus. There also was a moderate shrinkage of the middle and posterior insular cortex and superior temporal gyrus (**Figure 4**). Some remnants of the AVM were seen involving the left middle and superior frontal cortices [pre-supplementary motor area (pre-SMA) and SMA].

**FIGURE 5:**
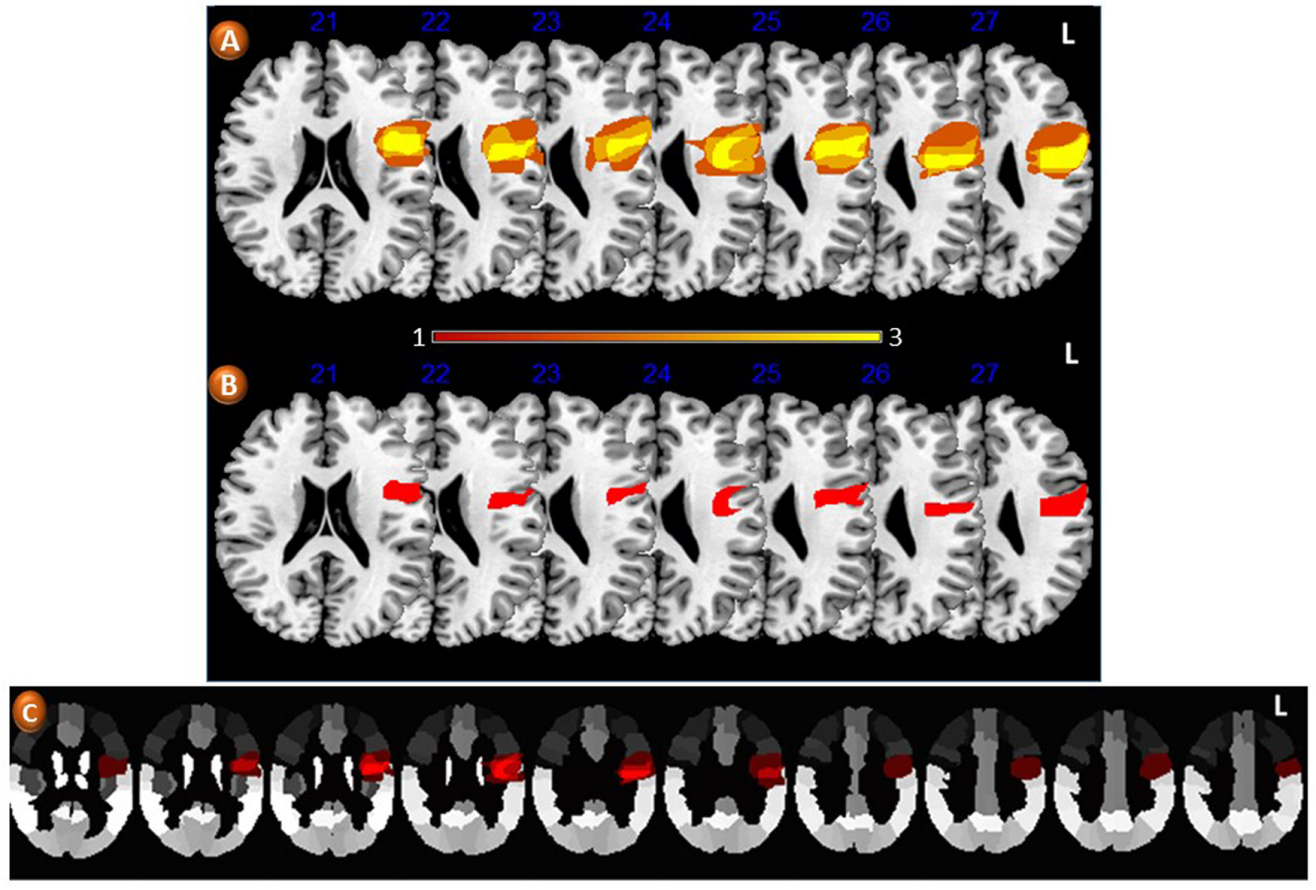
**Axial slices from T_1_-weighted MRI images in Montreal Neurological Institute space showing lesion overlap in three patients with loss of regional accent (A).** The average lesion density shows involvement of pericentral areas which are considered key components of the large-scale network mediating speech production. The color bar below **(A)** indicates the number of patients contributing to the average lesion image in both groups. **(B)** Shows the greatest area of lesion overlap in all three patients involving the medial part of the left precentral gyrus (red). **(C)** Depicts the areas of lesion overlap superimposed on the Automatic Anatomical Labeling (AAL) atlas (Tzourio-Mazoyer et al., 2002). L: indicates left.

Lesion overlap in the left pericentral region showed involvement of the inferior frontal gyrus (pars triangularis and pars opercularis), Rolandic operculum, middle frontal gyrus, precentral gyrus, post-central gyrus, and dorsal insula (**Figure 5**). The greatest area of overlap was in the left precentral gyrus (**Figure 5**).

### Analysis of Accent

#### Patient OM

##### Acoustic analysis

The overall impression in this patient was that he did not display a severe disturbance in speech articulation. Nevertheless, the production seemed asystematic as a multiplicity of variants occurred in those particular consonants that show a degree of variability among speakers of different Spanish dialects. The Córdoba accent presents the aspirated variant of /s/ in intervocalic and preconsonantic positions. A voiced variant of /s/ is observed in some speakers mainly in the north of the province of Córdoba. Thus, this should not be regarded as an instance of atypical production. After the stroke, the following realizations of /s/ in both intervocalic and preconsonatic positions were observed in OM ([h], [s], [z], [š], and [𝜃]). Furthermore, he occasionally mispronounced /s/, confusing this sound with a low intensity [ʃ]. The variants of [ʒ] in this region are [ʒ] and [λ], but OM alternated the variants [λ], [j], [ʃ], and [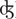], realizations that are found in other Spanish dialects. In several regions of Argentina (including Córdoba and Corrientes) the trill is realized as [ʒ] and in clusters this sound is produced as a voiceless fricative of brief duration. OM produced this sound as it is usually pronounced by typical residents of Córdoba. Healthy Spanish speakers produce the approximant voiced variants [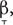 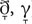] of the voiced stops /b, d, g/ in intervocalic position. Like some brain damaged patients with FAS, OM produced stops in this context, thus indicating poor regulation of co-articulation (Berthier et al., 1991). Vowel shifts are relatively frequent in the accent from Córdoba as well as from other areas of Argentina. In contrast, vowel shifts were not observed in OM, which may have contributed to the perception of regional accent loss. However, entire voiceless segments including vowels were distinguished in the spectrograms at the end of utterances, a finding that represents a misproduction. This phenomenon together with the occurrence of noise at 3000 Hz, may be indicative of poor control of phonatory vocal folding.

##### Prosodic analysis

**FIGURE 6:**
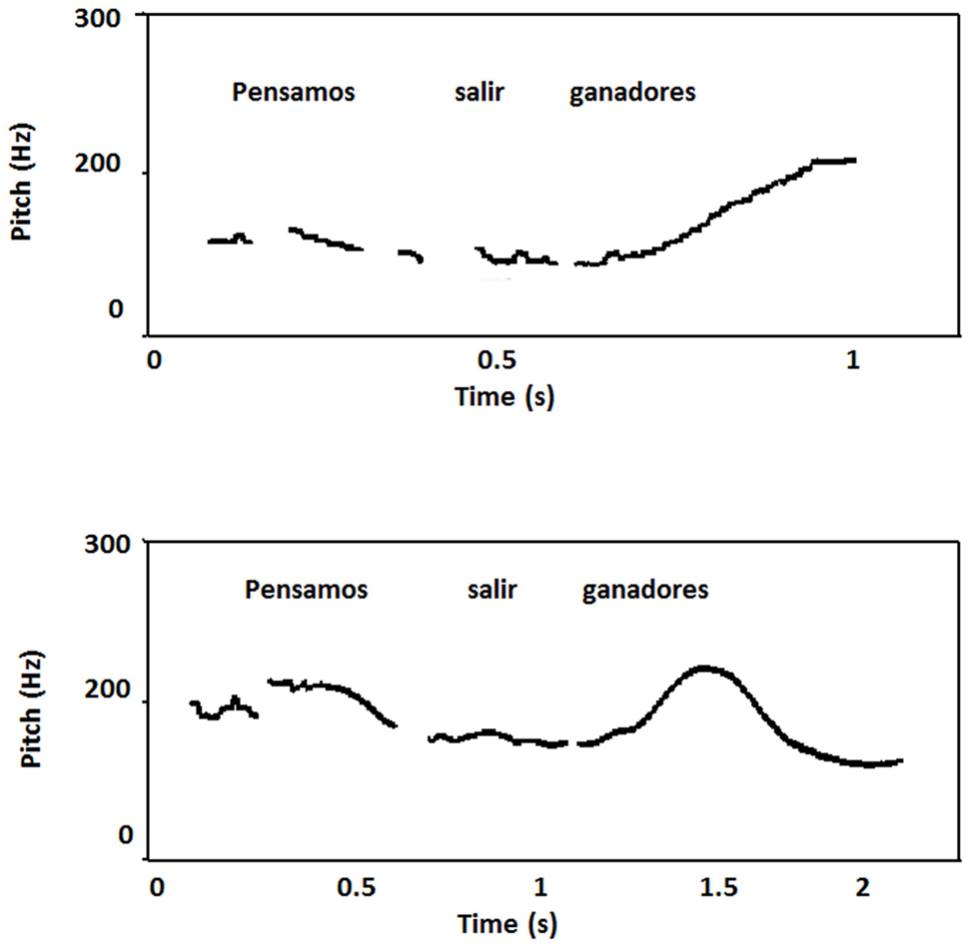
**Plots of the Spanish sentence “Pensamos salir ganadores” (“We aim to win”) before the stroke (top) and 8 months later (bottom) produced by patient OM.** The curve before the stroke shows a rising end contour. Note that F0 raises at the last accent syllable and continued to rise in the following unaccented ones. This seems to be a typical feature of Cordoba accent. After the stroke, there was a slow speech rate and analysis of F0 curve of the same declarative sentence showed a gradual declination. The slope of the top reference line presented an average rate of declination of about 41.4 Hz/s, similar to the value obtained in Spanish speaker from Buenos Aires (43 Hz/s).

Availability of comparable pre- and post-stroke samples of speech production allowed for an acoustic comparison, achieved by analyzing portions of OM’s speech recorded from one pre-stroke audiotape. The inspection of a few F0 curves obtained from OM in a recording previous to the stroke showed some characteristics that are presumably characteristic of Córdoba accent and deviate from other regions of Argentina. In effect, whereas in Spanish speakers with typical Buenos Aires regional accent the F0 contours present a general declining trend for both peaks and valleys in declarative sentences (Manrique and Signorini, 1983), the curves of OM showed a rising end contour; F0 raised at the last accent syllable and continued to rise in the following unaccented ones. The initial and final F0 average values were 138.3 ± 12.6 Hz and 195 ± 37.7 Hz, respectively. This phenomenon has been mentioned as “a kind of tonal shift” in previous studies of regional accent in Argentina. Average inter-stress interval duration was 447 ± 92.9 ms. Analysis of F0 curves after the stroke of the same declarative sentence (“Pensamos salir ganadores” “We aim to win”) showed a gradual declination. The slope of the top reference line presented an average rate of declination of about 41.4 Hz/s, similar to the value obtained in Spanish speaker from Buenos Aires (43 Hz/s). In yes-no questions overall F0 level was relatively high with regard to declarative sentences (average peak value in interrogative sentences: 152.5 Hz, final value: 288.8 Hz). However, this end rising pattern differed from the one observed in recordings obtained previous to the stroke for declarative sentences (**Figure 5**). In the latter, the overall level is lower (185 Hz). Otherwise, similarities were found in other F0 measures when pre- and post-stroke samples were compared (overall average F0: pre-stroke = 152.5 Hz; post-stroke = 155 Hz; range: pre-stroke = 80 Hz, post-stroke = 95 Hz; adjusted range: pre-stroke = 65 Hz, post-stroke = 54.02 Hz). However, these similar values in F0 range variation were due to high F0 values observed in the last portion of the utterances recorded previous to the stroke lesion. Except for this final portion of the utterances, the rest of the F0 contours showed a flat tendency with small F0 variation. Note that a fast speaking rate forced the F0 movements to be performed in less time and led to less F0 variation (O’Shaughnessy, 1976). Therefore, the fast speaking rate in the pre-stroke recording may explain the small F0 variation. Measures of temporal organization after the stroke showed a lower rate of speaking. Average overall duration of utterance was 20% longer than those obtained in the pre-stroke condition. Due to the fast rate of speaking observed in OM before the stroke, segment duration in the spectrograms was difficult to measure. Nevertheless, in the final portion of the utterances identified as having a rising F0 contour a lengthening effect of the entire syllable coincides with results previously reported for Córdoba accent (Fontanella de Weinberg, 1971). While average inter-stress intervals duration before the stroke were 447 ± 92.9 ms, duration was increased to 630 ± 250 ms after the stroke, an increment that can be explained by the insertion of pauses between words and the lack of co-articulation (**Figure 6**).

##### Prosodic tests

Interrogative sentences were accurately perceived by raters (100%). The sentences uttered with sad, angry, and happy intonation were mostly perceived as neutral (65% each) and mainly confused with sad. Sentences produced in neutral intonation were identified 92% as correct and confused (8%) with happy. When OM was asked to place tonic accent on some content words of a sentence from the perceptual test, his performance was flawless in all but one emission in which he emphasized every content word. The acoustic analysis showed increased F0 on stressed syllables with respect to the adjacent unstressed syllable. His comprehension of affectively intoned sentences was normal (94%).

#### Patient JF

##### Acoustic analysis

The patient’s speech production sounded similar to healthy Spanish speakers with no severe articulatory disturbances, although his production was different from the Guaranítico dialect, except in the case of the trill that continued to be produced as [ʒ]. The /ʒ/ is realized in Corrientes as [λ], [ʒ], or as [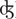], but several variations were found in JM including [λ], [ʒ], [ʃ], [j] and mispronounced as [li]. In the case of /s/ this fricative phoneme is normally aspirated in both intervocalic and preconsonantic positions and the /𝜃/ phoneme frequently appears in closed syllables. The patient produced either [s] or [h] in the positions mentioned. Moreover, his realizations of [l] were grossly under-articulated and the tongue failed to reach its normal target area in most occurrences. In Spanish, vowels are either oral or nasalized in nasal context and JF frequently produced normal oral and nasal vowels. Interestingly, there were similarities with the case of OM as there were voiceless segments at the end of utterances and noise bands located at 3900 Hz, also probably due to the lack of phonatory regulation.

##### Prosodic analysis

**FIGURE 7:**
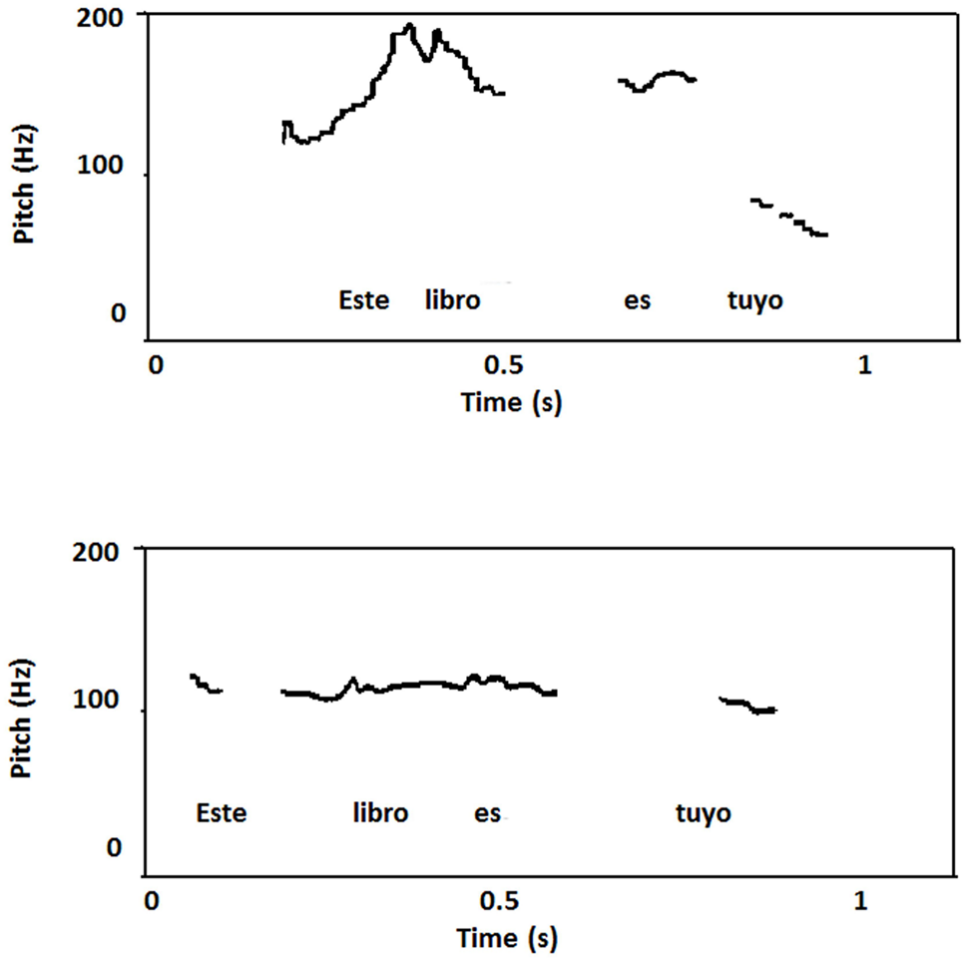
**Plot of the Spanish sentence “Este libro es tuyo” (“This book is yours”) pronounced by a healthy control subject with typical Corrientes’s accent (top) and patient JF (bottom).** The healthy control showed a higher rate of declination than patient JF who underwent small rise and fall movements giving a flat contour with some resetting effects. Limited movements of the F0 curve most likely contributed to the impression of a flat intonation.

There are no studies available of Guaranítico’s regional accent. Therefore, speech production samples from JF were compared with those obtained in a well-matched healthy control subject, and this comparison revealed important differences that seem to demonstrate a loss of regional accent in JF. In fact, the range of intra-speaker F0 variations in both subjects was different showing that in the case of JF the F0 had less range of rise and fall movements (range = 34.1 Hz, adjusted range = 26.3 Hz) compared to the control subject (range = 90.8 Hz; adjusted range = 48.4 Hz). These results in JF are consistent with those obtained in speakers of other languages after damage to the left hemisphere (Ryalls, 1982, 1984). The tracing of the top reference line in the F0 curves of both subjects showed a difference in the slope with the control subject presenting a higher rate of declination (34.6 Hz) than in JM (10.6 Hz). Thus, the general decline trend was much lower and more gradual in JM than in the control subject and his curves showed a flatter tendency with some resetting effects (the declination line resetted to a higher F0 level). This phenomenon most likely contributed to the perception of a monotone speech. Spectrograms and F0 curves showed that the overall duration in JM (1.85 s) was similar to that of the control subject (1.93 s), but there was a considerable amount of variability in the average duration on inter-stress intervals between JM (700 ± 170 ms) and the control subject (570 ± 60 ms). It was apparent that JM showed less segment duration and hence a tendency to isosyllabicity than the control subject who manifested a rhythmic pattern based on stress alternation (**Figure 7**).

##### Prosodic tests

Raters accurately identified most interrogative and neutral sentences (93%), but sentences intoned with happy, sad, and angry emotion were identified most commonly as neutral (57, 65, and 82%, respectively). Happy and sad intoned sentences were rarely perceived as correct (26%). The patient performed correctly when asked to place tonic accents. Measurements of F0 peaks in all content words showed relatively higher values on lexically stressed syllables of the target stimuli. His comprehension of affectively intoned sentences was normal (94%).

#### Patient RC

##### Acoustic analysis

The patient did not show prominent articulatory deficits, yet his regional accent was different to the regional accent of Buenos Aires. The different variants of preconsonantic /s/ constitute a peculiar aspect of Buenos Aires Spanish (Manrique, 1980). The most frequent realization of /s/ in this position is the voiceless glottal fricative [h]. However, RC produced the dental [s] in most contexts. Moreover, due to his socio-dialect the variant expected should be the [h] and not [s]. While Buenos Aires Spanish presents a short trill sound [r] with only one period in closed syllables (Massone, 1988), RC instead produced a longer trill with more than one period, a realization characteristic of CV syllables in Buenos Aires Spanish. As already indicated for patient OM, voiced stops are realized as approximants in intervocalic position (a feature common in the majority of the Spanish dialects). In this context as well as in initial position the stopping of approximants was produced indicating the absence of co-articulation. RC also had a frequent occurrence of voiceless segments both in final and medial position of words and utterances, lack of co-articulation, inappropriate pauses, and vowel lengthening. All these impairments denoted the lack of phonatory regulation of vocal folding.

##### Prosodic analysis

**FIGURE 8:**
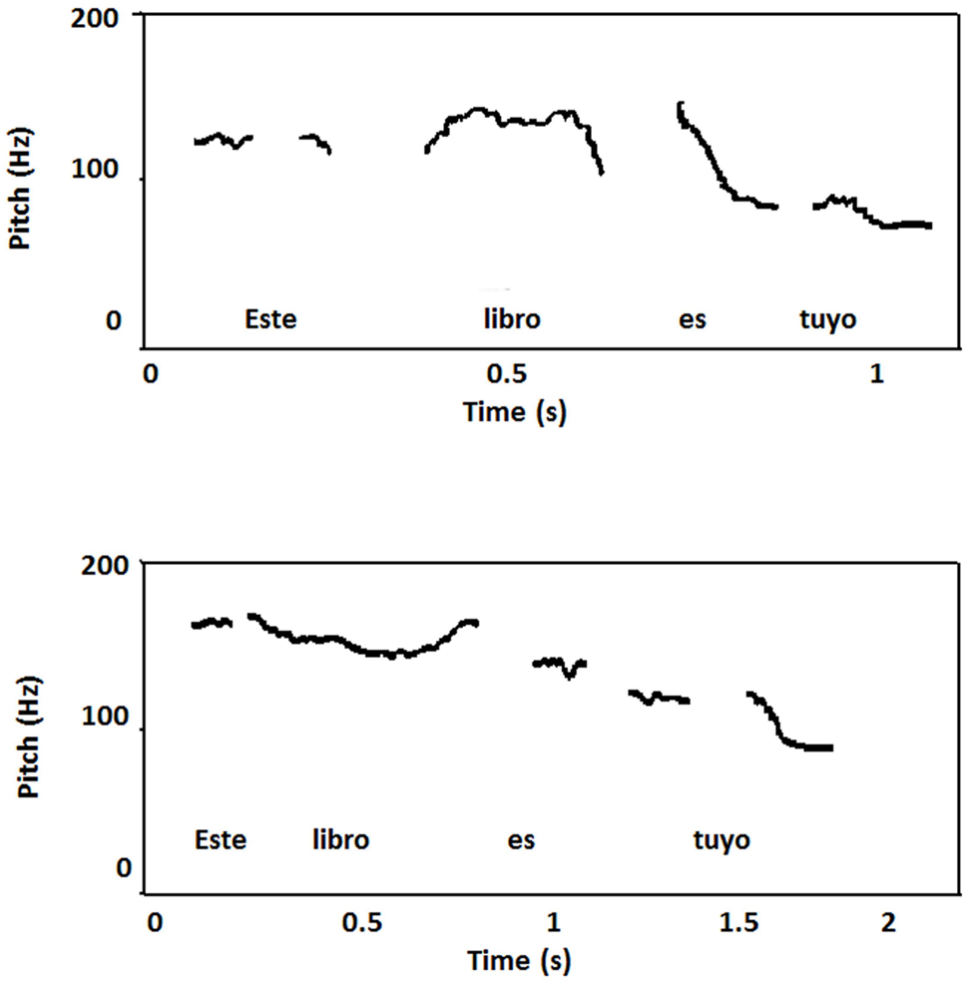
**Plot of the Spanish sentence “Este libro es tuyo” (“This book is yours”) pronounced by patient RC (top) and one of the healthy controls with typical Buenos Aires’s accent (bottom).** RC a slow speaking rate in part due to the insertion of inappropriate pauses. Curves showed less declining trend than healthy controls and in the in the last portion of the utterances F0 he continued to rise even in unaccented syllables.

Data obtained in RC were compared with findings on intonation and rhythmic patterns of Buenos Aires Spanish described in four healthy subjects (Manrique and Signorini, 1983; Massone et al., 1983). The range of intra-speaker F0 variation was different when compared with compared with RC (healthy controls: 64.3 Hz; RC: 82 Hz), but the adjusted range was similar (healthy controls: 42.7 Hz; RC: 43.6 Hz). However, the slow speaking rate of RC may account for the high range of F0 variation, since a slow rate allows sufficient time for all linguistic F0 movements. Inspection of the curves of declarative sentences through the tracing of the top reference line showed a declining trend (23.2 Hz/s) in RC which was considerably lower than observed in healthy controls (43 Hz/s). Taking into account that the range of F0 variation in RC was close to normal values, the fact that the top line did not reflect a steeper slope may be due to the slow speaking rate and also to the presence of numerous pauses that increased the utterance’s overall duration. Abnormal pauses may also be responsible for the length and variation observed in average duration of inter-stress intervals (RC: 820 ± 360 ms; healthy controls: 447 ± 92.9 ms; Manrique and Signorini, 1983). In healthy control subjects the production of interrogative yes-no questions had an overall F0 level that was relatively high with regard to declarative sentences, and a rising end contour. By contrast, this production in RC differed from normal because in the last portion of each declarative sentence F0 continued to rise even in unaccented syllables (**Figure 8**).

##### Prosodic test

Interrogative sentences were accurately identified (100%). Most sentences (83%) intoned with angry, happy, sad, and neutral emotions were identified as neutral, while a low percentage (14%) of sentences intoned with angry, happy, and neutral emotions were consistently confused with sad. The patient had problems to signal tonic accent in 22 out of 25 sentences. All content words in these sentences were perceived as bearing accent. In fact, the inspection of the curves showed F0 peaks in these words. In the only three correctly perceived sentences every content word also presented a F0 peak, but the presence of a pause before the portion of the utterance which should receive accent may account for this correct perception. His comprehension of affectively intoned sentences was normal (100%).

## Discussion

In the present case series study, we described loss of regional accent after selective damage of different components of the distributed neural network for speech production. Our patients were Argentinean monolingual speakers with three different native Spanish accents (Cordobés or central, Guaranítico or northeast, and Bonaerense). After suffering unilateral or bilateral focal lesions these patients presented with regressive Broca’s aphasia. Recovery of fluent production of speech was rapid (weeks) and it probably occurred because short fibers (subcallosum fasciculus; Naeser et al., 1989) and long-distance white matter tracts (arcuate fasciculus; Wang et al., 2013; Basilakos et al., 2014) previously linked to the persistence of reduced fluency were not affected. Regaining verbal communication and disappearance of typical features of Broca’s aphasia (agrammatism), however, did not assure in these patients the production of normal speech in that velocity and rhythm were slow and phonetic features characteristic of premorbid regional accents were attenuated or appeared in a random way. By the time of formal evaluation none of the patients could be classified as having aphasia since on the WAB they obtained an AQ well above the cut-off score (>93.8) for this diagnosis (Kertesz, 1982). Likewise, their performance on the TT, a difficult test of auditory comprehension which is useful to identify even very mild aphasic deficits (De Renzi and Vignolo, 1962), was also within normal limits. Reading, writing, or both were mildly impaired in two patients (OM and RC) but testing of general cognition (verbal and non-verbal intelligence, and praxis) was within normal limits. Samples of speech production were analyzed by expert phoneticians and linguists. Analysis of speech production revealed discrete slowing in speech rate, inappropriate long pauses, and monotonous intonation already described in FAS (Blumstein et al., 1987). Phonemic production remained similar to those of healthy Spanish speakers, but some phonetic variants distinctive to each accent (e.g., intervocalic aspiration of /s/ in Cordoba accent) were not present in any case. While basic normal prosodic characteristics of Spanish prosody were preserved, features intrinsic to melody of certain geographical areas (e.g., rising end F0 excursion in declarative sentences intoned with Córdoba accent) were absent. All patients managed to produce linguistic contrasts in sentences, but were impaired when producing affective prosody, a fact that rendered their speech monotonous. Brain imaging disclosed focal left hemisphere lesions (two hemorrhages and one abscess) mainly involving the middle part of the premotor/motor cortex with variable extension into their adjoining inferior and superior regions. The post-central cortex, posterior inferior and/or middle frontal cortices, anterior insula/putamen and SMA were also involved. Our findings suggest that lesions affecting the middle part of the left motor cortex and adjoining regions disrupt neural processes implicated in the production of regional accent features.

### Loss of Regional Accent or Foreign Accent Syndrome?

One intriguing characteristic of these three patients is that, while their brain lesions mostly overlapped with those of many previous patients with FAS (Berthier et al., 1991; Takayama et al., 1993; Sakurai et al., 2015), their accent was not perceived as foreign. On the contrary, they were perceived as native Argentinean speakers who did not have the typical regional accent. Two possibilities might be considered to explain this situation. One is that the linguistic characteristics of this population are fundamentally different from those of typical FAS patients. The other possible explanation is that, even if they share some of the linguistic characteristics described in FAS patients, the saliency of their specific errors produced the effect of dialectal change.

A close inspection of the data from the three patients reveals that they did have some of the characteristics of FAS patients. Most importantly, the slower speaking rate which was observed in the three patients had a negative impact on the speech rhythm and was possibly associated with F0 disturbances. In the case of JF and RC we also found a tendency to produce approximants as stops. This phenomenon implies that, similar to other FAS patients, the participants in this study did not produce the lenition processes which are characteristic of native speakers. In sum, the speech of these patients was not fundamentally different from the speech of other FAS patients. Thus, we should explain what made other speakers consider them to be Argentinean speakers without regional accent. It is important that none of the above mentioned characteristics (e.g., slow speech rate, flat F0, and stopping of approximants) is distinctive of any Argentinean (or Hispanic) dialect. This suggests that other characteristics of their speech may have blurred the effect in these three patients. It seems relevant that the three patients made errors in the production of a group of consonants that are possibly the most salient characteristic of Argentinean dialects (e.g., / [ʒ], ʃ/), as opposed to other Spanish dialects (e.g., Peruvian, Mexican, European). Note also, that while there are minor differences among the different Argentinean dialects, they all share some of these sounds. The fact that these patients retained some of these sounds, but the actual distribution did not coincide with that of their own dialect, may explain that they were perceived as Argentinean speakers who had lost their original accent.

It is highly conceivable that persons with FAS attenuate the segmental and suprasegmental features that characterize their native (regional) accent and additionally acquire deviant speech features that give rise to the impression of a person speaking with a foreign accent. Bearing in mind such complex phenomena, the question that now arises is whether our patients had lost their regional accent or whether they had developed speech changes consistent with a foreign accent. Moreover, another still unaddressed issue is whether one condition can evolve into the other one or whether the two conditions can occur simultaneously insofar as two patients (OM and JF) had moments of speaking with foreign accent preceding or coexisting with loss of regional accent. Normal human language is not fixed, uniform, or unvarying (Akmajian et al., 2010) and performance in language tasks among brain damaged individuals often fluctuates nearly in an hour-to-hour basis and also during the resolution of deficits. According to the testimonies volunteered by patients’ relatives, foreign accent was transiently perceived during the early recovery period in JF, but intriguingly it was heard for the first time in OM several months after onset when speech deficits are expected to be more stable. Before moving forward on this issue, it should be noted that the instances of foreign accent in our patients were based on the subjective impression of naïve listeners (patients’ relatives). In the case of patient OM, while attending the funeral of his father, some relatives perceived that he was speaking with a foreign accent resembling the one used by his Italian father. This episode in OM could be reminiscent of previous cases classified as “reversion to a previously learned foreign accent” after brain damage (Seliger et al., 1992; Roth et al., 1997). Healthy children overhearing a foreign accent can automatically imitate its prosodic pattern (Nakazima, 1962) and persons with extensive exposure to a different accent spoken by their parents could adopt the accents of their families after suffering damage to the speech production network (Seliger et al., 1992; Roth et al., 1997). For example, a left-handed woman with premorbid accent from “Bronx” or “New York” developed an Irish brogue (spoken by her mother many years ago) after suffering an infarct in left centrum semiovale underlying the parietal cortex (Seliger et al., 1992). A citizen of the United States with typical American English developed a Dutch accent after a left parietal hemorrhagic stroke (Roth et al., 1997). It was noteworthy that this patient was born in the Netherlands and lived there until the age of 5 years. Thus, awakening of a dormant foreign accent should be entertained in OM case. Nevertheless, the production of an Italian accent by OM was a short-lived phenomenon (hours) that came about during a stressful life event (bereavement). Thus, an emotional reaction accounting for an evanescent foreign accent could not be discarded (Van Borsel et al., 2005; Verhoeven et al., 2005). In the case of patient JF, his mother and an aunt reported that during the early recovery phase from aphasia, he spoke with a foreign accent resembling Japanese. This lasted a few days and afterward, according to the same relatives, JF’s speech was “flat” to the extent that “when he volunteered a question it was not possible to know if was actually asking something or not.” Assuming that both patients actually had instances of foreign accent, these were short-lived phenomena and these features were no longer present at the moment of formal linguistic evaluation. By contrast, loss of regional accent was a long-lasting disorder in OM and lasted at least 4 months in JF (he was lost for follow-up). Moreover, at the time of formal evaluation of speech production, three experts with knowledge of Italian (the Italian population in Argentina is the third largest in the world) did not find linguistic elements of such accent in OM’s verbal productions. Although the same three experts did not find hints of foreign accent in the other two patients (JF and RC) they concurred that the patients’ accents sounded “neutral” and devoid of the typical features of their respective regional accents.

### Neural Mechanisms

The interaction of different brain areas mediating speech production and monitoring is complex and, at present, is best conceptualized in the context of computational models like DIVA (Directions into Velocities of Articulators – Guenther et al., 2006) and GODIVA (Gradient Order DIVA – Bohland et al., 2010). These models of speech production control have been mapped onto anatomical regions in the human brain and their dysfunction has been invoked to explain FAS (Golfinopoulos et al., 2010; Tomasino et al., 2013). The role of brain regions involved in loss of regional accent will be briefly outlined in each of the next sections.

#### Motor, Premotor, and Sensorimotor Cortices

From a neuroanatomical viewpoint one relevant finding of the present study is that our three patients had damage to different components of the bilaterally distributed neural network mediating speech production. Analysis of lesion topography in our patients disclosed overlap along the central sulcus involving the medial part of primary motor cortical region [Brodmann’s areas (BAs) 4 and 6] with extension to its adjacent post-central cortex (BA 3), Rolandic operculum, middle frontal gyrus (BA 6), inferior frontal gyrus [pars triangularis (BA 45) and pars opercularis (BA 44) and dorsal insula (BA 13)]. The only area consistently involved in all three patients was the left precentral gyrus. Recent studies using brain imaging, transcranial magnetic stimulation, and computational modeling have provided empirical evidence of the role played by different regions of the speech production network. The initiation and planning of speech, the control of the articulators, and the monitoring of one’s own voice depends on the concerted activity of the primary motor and somatosensory cortices, auditory cortical areas, SMA, the precentral gyrus of the insula, and portions of the thalamus, basal ganglia, and cerebellum (Dronkers, 1996; Wise et al., 1999; Riecker et al., 2005; Sörös et al., 2006; Bohland and Guenther, 2006). Neuroimaging and brain stimulation studies reliably show that the activity elicited by both speech production and movements of the speech effectors is somatotopically organized with a dorso-ventral distribution (lip, jaw, vocalic/laryngeal, and tongue movements) in the motor cortex displaying an overlapping arrangement (Brown et al., 2008; Takai et al., 2010; Grabski et al., 2012) with great variability in the locations of activations among studies (see Brown et al., 2009 for a meta-analysis of phonation studies). This would imply that discrete damage to the primary motor cortex can induce different alterations of speech production (Eickhoff et al., 2009) and in the present cases it may have disrupted stored feedforward speech motor commands of phonetic features signaling regional accent (see Guenther et al., 2006). Indeed, circumscribed damage to the left primary motor cortex has been associated with different types of FAS affecting prosody (Takayama et al., 1993; Carbary et al., 2000; Katz et al., 2012), phonetics (Schiff et al., 1983; Kurowski et al., 1996), or both (Blumstein et al., 1987; Berthier et al., 1991; Scott et al., 2006). However, in the three patients described herein involvement of the middle part of the left primary motor/premotor cortices and their immediately adjacent areas did not induce FAS (but see below) as in the aforementioned cases with lesions restricted to these regions (Berthier et al., 1991; Takayama et al., 1993; Sakurai et al., 2015).

Since the cortical motor system is organized in a somatotopic fashion, features of articulatory/phonatory activities are controlled by different parts of motor and premotor cortex (Hauk et al., 2004; Pulvermüller et al., 2006). Previous brain imaging studies showed that the area with strongest activation in speech tasks corresponds to the region in the motor cortex underlying vocal folds activity (the larynx area; Brown et al., 2008; Simonyan, 2014). This cortical region has been repeatedly implicated in the pathogenesis of FAS (Berthier et al., 1991; Takayama et al., 1993; Tomasino et al., 2013: Sakurai et al., 2015). In one study, the left larynx area increased its activity to compensate the involvement of other areas relevant for phonation/articulation (Tomasino et al., 2013). By contrast, very discrete damage to activity of the larynx area correlated with abnormal prosodic production (Takayama et al., 1993) and prolongation of silent intervals between words (Sakurai et al., 2015) in previous FAS cases. We found that our three patients produced entire voiceless segments including vowels at the medial (RC) and final (OM and RC) position of words and utterances, a finding that reflects poor control of phonatory vocal vibration (Sakurai et al., 2015) most likely due to involvement of the larynx area in the left motor cortex.

This discrepancy between our patients and previous cases of FAS lies on the fact that the consistent damage to the left primary motor cortex in all three patients coexisted with involvement of other regions (insula, striatum, pre-SMA, and SMA) which were not simultaneously damaged in previous cases of FAS. Patient OM had a small hemorrhage involving the anterior insula/putamen region with minimal dorsal extension into the inferior frontal cortex, yet this lesion was right-lateralized and not placed in the left hemisphere as was described in previous cases of FAS (Scott et al., 2006; Moreno-Torres et al., 2013). By contrast, focal insular involvement in RC was in the left hemisphere and occupied in the anterior sector and extended into the anterior putamen. The rest of the left insular cortex together with ventral premotor and motor cortices, and superior temporal gyrus showed post-stroke atrophic changes. In patient JF, the cerebral abscess impinged upon the left anterior insula and displaced it medially but these pressure effects were no longer detected in a post-surgical CT which disclosed only a small residual lesion in the left primary motor cortex. The role of involvement of these cortical and subcortical regions on accent change is described below.

#### Insula

The insula acts as a multimodal integration hub to coordinate the activity of number of regions important for verbal and non-verbal processing (Mesulam and Mufson, 1985; Habib et al., 1995; Ackermann and Riecker, 2004, 2010). Moreover, the left anterior insula is a key component of the planning network for speech production playing a role in the formulation of complex articulation (Wise et al., 1999; Ackermann and Riecker, 2004, 2010; Riecker et al., 2005; Baldo et al., 2011). It is also important for producing speech with a distinct rhythm/intonation structure (Scott et al., 2006) and for phonetic learning (Golestani et al., 2007; Ventura-Campos et al., 2013). Insular involvement alone (Scott et al., 2006) or associated with damage to other regions (Moreno-Torres et al., 2013) has been described in FAS. Nevertheless, insular involvement in our cases did not induce FAS but it may well have contributed to an alteration of regional accent characteristics by causing additional deficits in the production of emotional prosody and, to a lesser extent, of linguistic prosody. In fact, by virtue of its strong connections with limbic and paralimbic regions, insular damage may play a key role on adjusting motor speech to speaker’s emotional status (Mesulam and Mufson, 1985; Ackermann and Riecker, 2004, 2010). Insular damage in OM and RC or dysfunction in JF would have reduced modulation of prosody particularly in emotional contexts, eventually leading to a monotonous emissions (Ackermann and Riecker, 2004, 2010). Monotonous speech along with attenuation of phonetic features of native (regional) speech may have greatly influenced the opinion of experts’ judges to classify speech production as lacking regional accent discarding the diagnosis of FAS.

#### Putamen

The right and left anterior putamen were involved in OM and RC, respectively. The role of the left putamen in speech articulation is supported by cases of FAS (Gurd et al., 1988) and by emergence of regional variant of her native language (Naidoo et al., 2008) both occurring after lesions restricted to it. Stroke lesions close to the left putamen have also been associated with loss of regional accent in a single patient (Alexander et al., 1987), whereas a loss of premorbid talent to imitate several dialects followed a stroke lesion in the right putamen (patient 2 in Van Lancker Sidtis et al., 2006). The putamen is part of the cortico-striato-thalamo-cortical network (Alexander et al., 1986) and acts as a relay station between the pre-SMA and motor cortex, so that its damage may alter the interplay between planning and execution of speech motor acts.

#### Pre-SMA and SMA

The pre-SMA is related to linguistic processing (Catani et al., 2013), whereas the SMA proper participates in speech initiation, coordination and speech monitoring (Laplane et al., 1977; Crosson et al., 2001; Alario et al., 2006). Both, the pre-SMA and SMA play a role on planning and motor initiation and interact with the executive motor cortex via the basal ganglia (motor loop) and thalamus (Bohland and Guenther, 2006; Bohland et al., 2010). Lesion mapping studies show that damage to medial frontal cortex (pre-SMA and SMA) interrupting (or not) the frontal aslant tract (FAT) has been associated with speech arrest (Martino et al., 2012), reduced speech fluency (Catani et al., 2013; Basilakos et al., 2014; Kronfeld-Duenias et al., 2014), and impaired morphological derivation of verbs (Sierpowska et al., 2015). The left pre-SMA and SMA were affected in patient RC and its involvement most likely disrupted connectivity through U-fibers with the precentral cortex and cingulate cortex and through long-distance tracts with the striatum and pars opercularis of the inferior frontal cortex via the FAT (Vergani et al., 2014). Involvement of the left SMA may have also interrupted in RC connectivity with the left Heschl’s gyrus (both cortical areas were damaged) altering monitoring during overt speech production (McGuire et al., 1996; Christoffels et al., 2007; Toyomura et al., 2007; van de Ven et al., 2009) and hence contributing to RC’s lack of insight about his accent change. On the other hand, he was fully aware of the flatness of his emissions presumably because the right SMA and Heschl’s gyrus needed for informing the speaker on the status of emotional prosody production remained functional. Thus, involvement of the left pre-SMA and SMA was in a position to disrupt their dynamic interplay with structures important for planning and execution of speech as well as speech monitoring.

However, the contribution of the left pre-SMA, SMA and the origins of FAT to symptomatology in patient RC should be interpreted with caution. These structures were invaded by the AVM from early brain development, thus suggesting reshaping of original functions in other cortical regions (Lazar, 2001). Even though the left primary motor cortex was not involved by the AVM, the fact that handedness had changed might have modified its somatotopic arrangement (Klöppel et al., 2007, 2010) perhaps influencing the effects of brain damage. There are some clues that brain reorganization of language could have taken place in RC. First, he was a forced left-hander, a condition defined as “innately left-handed individuals forced to write with the non-dominant right hand.” It has been demonstrated that being a forced left-hander modifies the architecture of the primary motor cortex (Siebner et al., 2002; Klöppel et al., 2007, 2010) thus implying that damage to such region in the left hemisphere might not exerted the same deficits as reported in right handed individuals. Second, RC showed a surprisingly rapid and complete recovery of aphasia despite having involvement of the left central perisylvian language cortex. Third, despite having a left hemisphere lesion, RC’s performance on the WAIS was highly discrepant with unexpectedly higher verbal (111) than performance (97) IQ scores. Taken as a whole, these findings favors the position that some language functions were transferred to the right hemisphere during development (Vikingstad et al., 2000; Berthier et al., 2011) although some intra-hemispheric reorganization to regions adjacent to the medial frontal cortex cannot be excluded (Duffau et al., 2000). Early cross-hemispheric plasticity may have assured normal speech and language acquisition in RC (he had no history of speech-language disability) but possibly interfered with typical right hemisphere cognitive functions, the so-called “crowding effect” (Satz et al., 1994; Lidzba et al., 2006). Lazar et al. (2000) using functional MRI showed that one of their patients (case 2) with a left frontal AVMs had mirror reverse pattern of activation in the right hemisphere (insula, frontal operculum pars opercularis, and inferior frontal gyrus) during word-list generation.

## Conclusion

In summary, the results of the present case series study suggest that damage to the left premotor/motor cortex and other nodes of the speech production network (insula, basal ganglia, pre-SMA, and SMA) can alter segmental and suprasegmental features that characterize regional accents. Loss of regional accent was long-lasting in the two patients who had additional damage to other structures involved in speech production, thus suggesting that these lesions exerted an additive negative effect precluding full spontaneous recovery. Further studies using functional neuroimaging are required to examine more fully the potential contribution of dysfunction of different components of the speech production network to both the emergence as well as the persistence of accent change. Moreover, studies examining the impact of losing regional accent on functional communication and psychosocial adjustment are strongly needed (see Lippi-Green, 1994; Miller et al., 2011; Moreno-Torres et al., 2013). Finally, we suggest that loss of regional accent should be added to the spectrum of disorders characterized by changes in accent after brain damage, like FAS and its variants.

## Author Contributions

MB, GD, IM-T, and MM were involved in conception and design, acquisition of data, or analysis and interpretation of data. RR-C, NV, and KT-H interpreted neuroimaging data. MB, AB-C, DS-M, and MT-P drafted the article and revised it critically for important intellectual content; and all authors approved the final version for publication.

## Conflict of Interest Statement

The authors declare that the research was conducted in the absence of any commercial or financial relationships that could be construed as a potential conflict of interest.
